# The impact of women’s education, workforce experience, and the One Child Policy on fertility in China: a census study in Guangdong, China

**DOI:** 10.1186/s40064-016-3424-6

**Published:** 2016-10-04

**Authors:** Manyu Lan, Yaoqiu Kuang

**Affiliations:** 1 Sustainable Development Research Center, Guangzhou Institute of Geochemistry, Chinese Academy of Sciences, 511 Kehua Street, Wushan, Tianhe District, Guangzhou, 510640 China; 2 University of Chinese Academy of Sciences, 19 A Yuquan Rd, Shijingshan District, Beijing, 100049 China; 3 South China University of Technology, 381 Wushan Road, Tianhe District, Guangzhou, 510641 China

**Keywords:** Fertility, Education, Workforce experience, One Child Policy, Guangdong

## Abstract

The impact of women’s education on fertility is of interest to researchers, particularly in China. However, few studies have provided well-founded assessments of how women’s education, workforce experience, and birth control policy jointly affect fertility in China. This study, conducted in Guangdong Province, aimed to analyze how these three factors influenced the timing of births and affected women at different stages of their reproductive lives. We used census data for Guangdong Province (1990, 2000, and 2010) to make cross-sectional age-specific comparisons to examine the effects of women’s education and workforce participation on fertility outcomes under China’s One Child Policy. We found that: (1) under circumstances of low fertility, women tend to have more children with greater educational attainment; (2) the impact of women’s education and workforce experience on fertility varied across age groups, with the effect of education showing a bimodal curve peaking at 25–29 years and 40–44 years, and a workforce experience effect at 25–34 years; and (3) the fertility time-squeeze effect by educational attainment was relatively small, the effect by workforce participation was larger, and the most important effect was birth control policy and its implementation. These results suggest that educational attainment and workforce experience have a substantial effect on women’s fertility, and a tradeoff between them is unavoidable. China’s 2015 birth control policy adjustment should be considered in planning future services to accommodate anticipated increases in the birth rate. More attention should be directed to the causal mechanism (women’s preference and selection effects) behind the factors analyzed in this study.

## Background

From the early 1990s, the total fertility rate (TFR) in China dropped to below replacement level (TFR of 2.1) as a result of aggressive birth control programs (Zhao and Zhang 2010). China’s population census in 2000 reported the TFR was 1.22; fertility in 2010 was even lower (TFR of 1.18) (NBSPRC 2002, 2012). By design, China has experienced a long period of low fertility. At the same time, there has been a substantial increase in the length of compulsory education, which contributed to improvements in the education level of the population overall. In Guangdong Province, China, compulsory education for women increased from 9 to 12 years. As in the rest of China, fertility in Guangdong has continued on a downward trend, which is cause for social concern. It is suggested that birth control policy, women’s education, and women’s workforce participation are key factors affecting fertility in China (Rindfuss et al. 1996).

The specificity of Chinese birth control policy is a key contextual factor. In the face of burgeoning population growth, China implemented the One Child Policy in 1979 to decrease the fertility rate. This policy limited couples to one child. Couples who violated this restriction were subject to fines and other penalties. For most Chinese women, this strict policy resulted in one child (or no child). Educational attainment has been identified as another major factor influencing women’s postponement of childbearing (Marini 1984), and has received considerable research attention. In contrast, the consequences of women’s workforce participation, another important factor, have received less attention. Schultz and Zeng (1995) found that women’s educational attainment and the availability of health and family planning services significantly and negatively affected fertility in rural China. Rosenzweig and Zhang (2009) reported that the China’s One Child Policy lowered fertility, but improved the average quality of children. However, neither study examined the relationship between women’s workforce experience and fertility behavior.

Many empirical studies (Bloom and Trussell 1984; Rindfuss et al. 1996) have pointed to a robust negative association between women’s education and fertility. However, in recent years, this relationship has been the focus of increasing controversy from both theoretical and empirical perspectives. For example, an increasing number of studies have reported that women with higher levels of education have more higher order births in Northern Europe (Hoem and Hoem 1989; Kravdal 2007), Western Europe (Köppen 2006; Kreyenfeld and Zabel 2005), and Austria (Hoem et al. 2001). Before childbirth, women also have to consider the opportunity costs of childrearing, and are therefore sensitive to the timing of employment interruption (Gustafsson 2001; Taniguchi 1999). Evidence for a reduction in the negative time-series association between fertility and female workforce participation was found among the Organization for Economic Cooperation and Development member countries after about 1985 (Kogel 2004). Some researchers have suggested simultaneous models including fertility, labor supply, and childcare factors. Studies have confirmed that women’s workforce participation reduces their parental childcare time, and childcare may also result in motherhood wage penalty (Craig 2007; Molina and Montuenga 2009). Women’s workforce participation is generally considered to contribute to the postponement of childbearing, but it is not clear if this delay leads to later births or to childlessness (Nicoletti and Tanturri 2008; Billingsley et al. 2014).

Most of the early literature on the relationship between fertility and education considered women as a whole, examining such factors as status, sibship, cohort, and district; few studies have considered women’s age groups. Women in various age groups may be affected differently in terms of postponement of childbearing and the number of children desired. These causal links seem plausible, but there are limited data supporting their relative importance. China’s One Child Policy is unique, and its constraints on childbearing are apparent and persistent. Therefore, more attention should be directed to factors other than educational attainment that affect childbearing and fertility in China, particularly workforce experience and age group differentials. As few studies provided well-founded assessments of how these factors jointly influence fertility in China, we explored the linkages between women’s fertility-timing behavior, educational attainment, and workforce experience. Although women’s reproductive behavior involves many socioeconomic and cultural factors, modeling these additional processes is beyond the scope of this paper.

## Methods

**Fig. 1:**
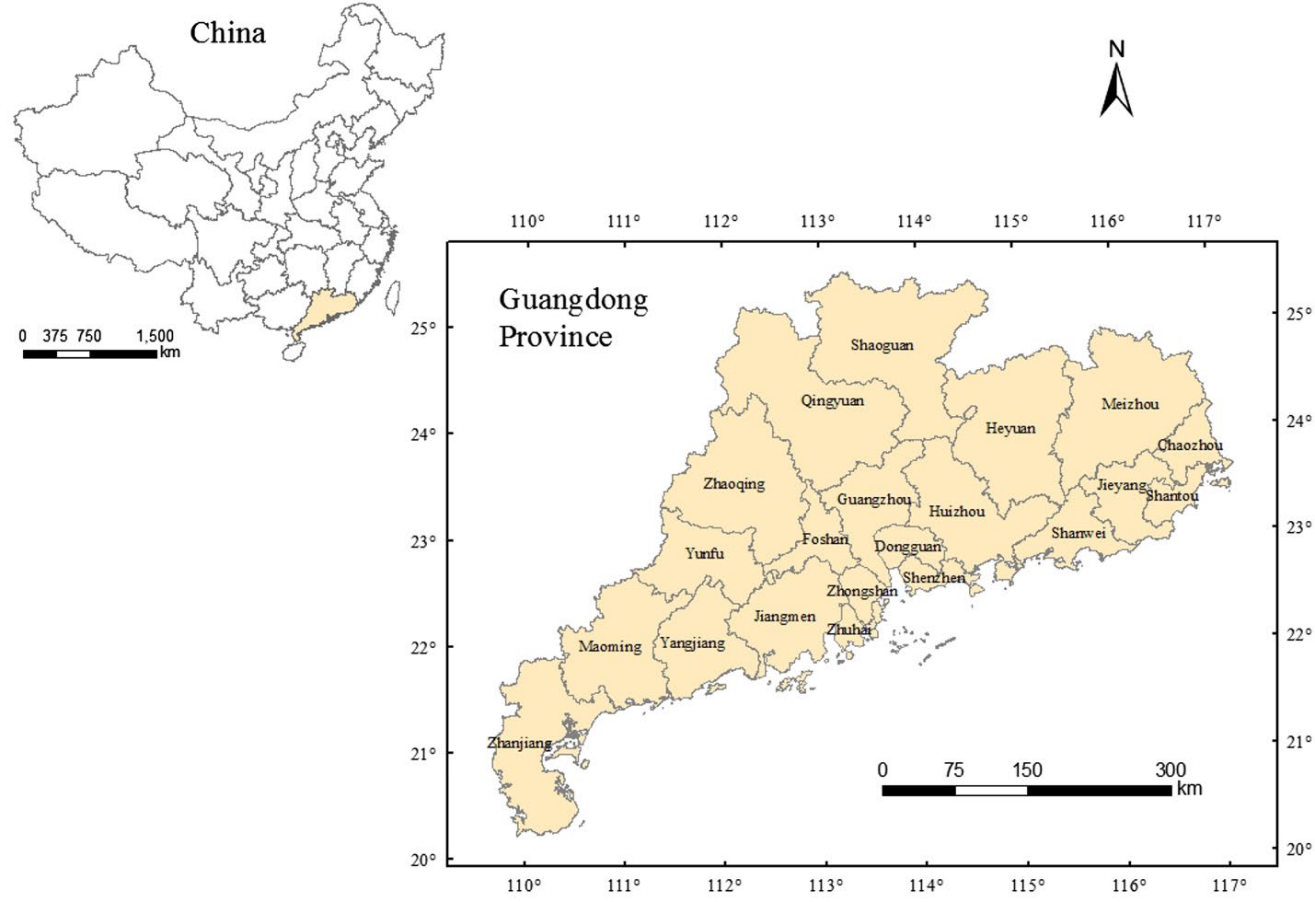
Location of Guangdong Province and its 21 administrative cities

We chose Guangdong Province as the focus for our study (Fig. 1), and used population census data for 1990, 2000, and 2010 in our analysis. Guangdong was selected for three reasons: First, it is the most densely populated province in China. The population was 104,303,132 people in 2010, accounting for 7.79 % of China’s total population. The population of the province is larger than that of Germany, or the three largest states in the United States (California, Texas, and New York). Second, in the 1990s, Guangdong’s fertility declined to below-replacement level. Third, Guangdong was the first province to implement the Reform and Opening Policy; that is, the Chinese market-oriented reforms to promote economic development. This means Guangdong has unique socioeconomic and geographic characteristics compared with other provinces in China. Since 1989, Guangdong has topped the total GDP rankings of all provinces, and contributes approximately 12 % of China’s national economic output. Regional perspectives may also help to improve understanding of the dynamics of postponement of childbearing and other aspects of reproductive behavior over time and at different paces. Moreover, the census data allowed tests for interaction between period trends and age patterns in fertility.

To assess the impact of women’s educational attainment, workforce experience, and the One Child Policy on fertility, we used a sample of women aged 15–49 years (i.e., women of childbearing age) drawn from each census year (1990, 2000, and 2010). We used a census-based cross-sectional analysis to establish an empirical framework. Control variables included educational attainment, workforce experience, and the effectiveness of the government-implemented birth control policy. We divided women into subgroups by age. The regression framework can be described as:

1 $$Child_{it}\, = \,\alpha_{0} \,+ \,\alpha_{1} Edu_{it} \,+ \,\alpha_{2} Exper_{it}\, +\, \alpha_{3} Expersq_{it} \,+\, \alpha_{4} Pol\, +\, \alpha_{5} Cohort_{t} \,+\, \alpha_{6} Edu_{it} \,\times \,Cohort_{it}\, +\, \varepsilon_{it} .$$

Here, *i* represents individuals, *t* represents census years. *Child* is the number of children born in *t* years, and *Edu* is the level of education completed by individual *i*. We used a close proxy of $\widehat{Child}$ to stand for *Child*. $\widehat{Child}$ denotes the cumulative fertility at the end of the census year: $\widehat{Child}$ = *Br*_*x*_/*Wm*_*x*_, where *Br*_*x*_ means number of children ever born to women in age group *x* and *Wm*_*x*_ means women who had ever given birth in age group *x*.

Educational attainment qualifications for *Edu* were disaggregated into seven levels: illiterate (less than primary school education completed), primary school completed, junior high school completed, senior high school completed, junior college or post-secondary certificate completed, undergraduate completed, and graduate completed. *Edu* was measured by average years of education (as constructed by Barro and Lee 2001), set to 3, 6, 9, 12, 15, 16, and 19. As an illiterate person may acquire knowledge through social learning (interpersonal communication) and observation, we evaluated this level as 3, rather than 0.

*Exper* refers to a woman’s workforce experience (number of years of labor force participation) in the census year before giving birth. This variable was designed to measure the level of incompatibility (in allocation of time) between workforce experience and childbearing (family building). To simplify the measurement of workforce interruptions, we adopted the expression of accumulating experience in the Mincer equation. This equation is a single-equation model that explains earnings as a function of schooling and experience. In the Mincer equation, educational attainment is summarized by years of education, with the potential work experience measured as Age − *Edu* − 6 (i.e., age minus education minus 6) (Mincer 1958, 1974). *Expersq* means the square of workforce experience (÷100). We regarded duration of workforce experience as an important variable in measuring the degree to which women’s fertility desires are suppressed; assuming that such fertility desires accumulate over time.

*Pol* denotes the effectiveness of the birth control policy implemented by local governments, and is measured by the proportion of out-of-quota births (i.e., the proportion of women with more than one child) in various age groups in that statistical year. Fertility has been strictly controlled in China, but there are out-of-quota births, especially in rural areas.

Changes over time are addressed in the analysis. We defined *Cohort* as dummy variables of childbirth cohorts (i.e., groups of women who gave birth in each of the census years). *Edu* × *Cohort* denotes the interaction of educational level and childbirth cohort, to observe the effect of education in different periods. ε is a disturbance term.

### Data analysis

**Fig. 2:**
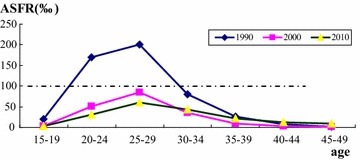
Age-specific fertility rates (ASFR) of Guangdong in 1990, 2000, and 2010

Figure 2 shows age-specific fertility rates (ASFR) by 5-year age groups in Guangdong Province for census years 1990, 2000, and 2010. The most obvious similarity of the three curves is that fertility peaked among women aged 25–29 years. However, age-specific fertility in 2000 and 2010 was much lower than in 1990, and the data support a steep decline in fertility. Using a reference standard of ASFR = 100 ‰, the fertility distribution in 1990 had an age span of over 10 years, while fertility in 2000 and 2010 was sharply below 100 ‰ and maintained a lower smooth curve. Compared with the 2000 cohort, fertility in 2010 was lower in women aged 15–29 years, but slightly higher in those aged 30–49 years. This difference was particularly clear in those aged 40–49 years, meaning that women’s fertility in 2010 had longer duration at older ages. The impact of the One Child Policy means that the age-specific fertility curve gradually flattened with each succeeding census, resulting in lower fertility peaks, later childbearing, and fewer children overall.

Overall, nearly 1 million women were included in our analysis: 777,065 in 1990, 76,654 in 2000, and 91,866 in 2010. The average number of years of schooling completed over this period increased substantially. Among the sampled women who gave birth, educational attainment increased from 7.4 years in 1990 to 8.8 years in 2000, then to 13.9 years in 2010. The number of children born by the end of the census year averaged 2.03 in 1990, 1.69 in 2000, and 1.38 in 2010. Enforcement of the One Child Policy appears to have been strengthened during this time, and the percentage of out-of-quota children decreased from 0.59 in 1990, to 0.30 in 2010. We used a crosstab analysis to examine the number of children, mother’s educational attainment, and mother’s career (occupational status). These were strongly correlated, with a two-tailed significance value of nearly 0. We found the same results using Pearson’s Chi squared test and the likelihood ratio test.

## Results

### Results for the total sample

Table 1 shows our estimates (regression coefficients) for all cohorts in 1990, 2000, and 2010 for the effects of women’s education, workforce experience, and the One Child Policy on women’s fertility. The coefficients of *Cohort*_*00*_ and *Cohort*_*10*_ were significantly negative, meaning the variations in fertility over time were more significant for the 2000 and 2010 cohorts than the 1990 cohort. For the 1990 cohort (*Cohort*_*00*_ = 0, *Cohort*_*10*_ = 0), the regression coefficient of *Edu* was −0.050 and was significant at 95 %. The coefficients of *Edu* × *Cohort*_*00*_ *, Edu* × *Cohort*_*10*_ were significantly positive, indicating that the coefficient of *Edu* was 0.021 for the 2000 cohort (*Cohort*_*00*_ = 1, *Cohort*_*10*_ = 0), and 0.030 for the 2010 cohort (*Cohort*_*00*_ = 0, *Cohort*_*10*_ = 1). These results show an interesting fertility pattern. In 1990 women with more education did not want more children, but in 2000 and 2010, women’s intentions changed. Under the circumstances of low fertility, women tended to have more children with the increase of education level, even constrained by the fertility policy.

**Table 1:** The average effect of female education, experience and policy on fertility

	$$\widehat{Child}$$
*(Constant)*	1.760*** (0.078)
*Edu*	−0.050*** (0.006)
*Pol*	2.376*** (0.063)
*Exper*	−0.048*** (0.005)
*Expersq÷100*	0.122*** (0.013)
*Cohort*_*00*_	−1.027*** (0.078)
*Cohort*_*10*_	−1.185*** (0.084)
*Edu* × *Cohort*_*00*_	0.071*** (0.007)
*Edu* × *Cohort*_*10*_	0.080*** (0.007)
Observations	686
Adj. R^2^	0.867

Standard errors are in parentheses. *,** and *** stand for significant at the 10, 5, and 1 % level, respectively

The regression coefficient of *Exper* was also significantly negative at a 99 % level. With the addition of workforce participation, fertility declined. A possible reason is that work experience reduced the fecund years available to women for reproduction. The effectiveness of the One Child Policy was calculated using the percentage of out-of-quota children born to various age groups. The regressions showed this was significantly positive, indicating that the more loosely the policy was implemented, the more children were born. The results of the One Child Policy are consistent with data from Guangdong.

### Results for age-specific subsamples

For the general estimates above we pooled all age groups, representing an average effect over time. However, there were notable differences among the age groups. We analyzed the age-specific effects of education and workforce participation on fertility to provide better insight into the differentials of fertility. We performed a multiple group comparison separately for each age group. We disaggregated the women into seven 5-year age groups: 15–19, 20–24, 25–29, 30–34, 35–39, 40–44, and 45–49 years, to focus on the effect of age on fertility behaviors. The 5-year age group was adopted as this interval is used by most statistical researchers (Bloom et al. 2009; Cai 2008). The results of the age-specific analyses are presented in Table 2. We used both standardized and unstandardized coefficients to facilitate comparison between coefficients and across time. The possibility that one direction dominated the overall relationship among education, workforce experience, and birth control policy can be evaluated by examining the magnitude of the standardized coefficients. The standardized coefficients showed that the main factors that affected fertility separately were: the One Child Policy at ages 15–34 and 40–44 years, educational attainment at age 35–49 years, and work experience at age 45–49 years.

**Table 2:** The age-specific effect of female education, workforce experience and birth control policy on fertility

$\widehat{Child}$	15–19		20–24		25–29		30–34		35–39		40–44		45–49	
	(1)	(2)	(3)	(4)	(5)	(6)	(7)	(8)	(9)	(10)	(11)	(12)	(13)	(14)
*(Constant)*	0.894*** (0.095)		0.958*** (0.056)		1.010*** (0.133)		2.383*** (0.199)		3.175*** (0.454)		1.282 (1.587)		4.838*** (1.755)	
*Edu*	0.006 (0.008)	0.091	0.003 (0.004)	0.060	0.003 (0.007)	0.030	−0.072*** (0.009)	−0.518***	−0.138*** (0.015)	−0.857***	−0.051 (0.039)	−0.164	−0.104*** (0.029)	−0.476***
*Pol*	1.523*** (0.074)	0.911***	1.256*** (0.056)	0.949***	1.841*** (0.043)	1.069***	1.488*** (0.085)	0.696***	1.373*** (0.113)	0.502***	3.338*** (0.132)	0.934***	1.662*** (0.209)	0.521***
*Exper*	0.019 (0.015)	0.231	−0.004 (0.005)	−0.080	−0.027*** (0.009)	−0.242***	−0.064*** (0.015)	−0.475***	−0.032 (0.029)	−0.210	−0.083 (0.094)	−0.279	−0.118 (0.099)	−0.580
*Expersq÷100*	−0.174 (0.119)	−0.202	0.055** (0.026)	0.165**	0.123*** (0.032)	0.246***	0.257*** (0.038)	0.608***	0.115* (0.065)	0.315*	0.257 (0.172)	0.449	0.187 (0.147)	0.580
*Cohort*_*00*_	−0.027 (0.059)	−0.046	0.040 (0.030)	0.087	−0.221*** (0.049)	−0.188***	−1.183*** (0.063)	−0.817***	−1.748*** (0.101)	−1.058***	−2.273*** (0.260)	−0.679***	−1.765*** (0.195)	−0.793***
*Cohort*_*10*_	−0.142** (0.061)	−0.235**	−0.023 (0.028)	−0.048	−0.303*** (0.054)	−0.244***	−1.351*** (0.069)	−0.879***	−2.166*** (0.108)	−1.262***	−2.229*** (0.277)	−0.680***	−1.988*** (0.207)	−0.937***
*Edu* × *Cohort*_*00*_	0.003 (0.007)	0.046	−0.003 (0.003)	−0.073	0.015*** (0.004)	0.162***	0.079*** (0.005)	0.686***	0.116*** (0.009)	0.824***	0.169*** (0.025)	0.527***	0.062*** (0.023)	0.238***
*Edu* × *Cohort*_*10*_	0.017** (0.006)	0.300**	0.001 (0.002)	0.015	0.016*** (0.005)	0.168***	0.088*** (0.006)	0.746***	0.150*** (0.009)	1.148***	0.168*** (0.024)	0.676***	0.087*** (0.023)	0.527***
Observations	115		115		115		115		115		115		115	
Adj. R^2^	0.925		0.960		0.977		0.978		0.951		0.917		0.910	

Standard errors are in parentheses. *,** and *** stand for significant at the 10, 5, and 1 % level respectively. Regressions 1, 3, 5, 7, 9, 11, 13 present unstandardized coefficients and 2, 4, 6, 8, 10, 12, 14 present standardized regression coefficients

**Fig. 3:**
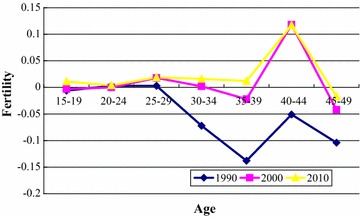
Effect of education on fertility for age-specific groups in 1990, 2000, and 2010

We examined relationships across age groups and cohorts to test whether sources of variation in fertility were the result of interaction. In 1990, the effect of education was not significant at age 15–29 years, but the education effects in the groups aged 30–34, 35–39, and 45–49 years were significant and negative. The coefficients of *Cohort*_*00*_ and *Cohort*_*10*_ were significantly negative, with the exception of the groups aged 15–19 and 20–24 years. This suggests that the time-varying effects were more marked in the later cohorts than in the earlier cohort, similar to the results for the total sample. We analyzed the interaction between education and cohort (i.e., *Edu* × *Cohort*_*00*_ and *Edu* × *Cohort*_*10*_) to determine the effect of education in different periods and age groups. We then calculated the age-specific effects of educational attainment on fertility. As seen in Fig. 3, the shapes of the curves for the three census years were similar, but the magnitude of the effects in 2000 and 2010 were markedly different compared with those in 1990. Although there were cohort effects, the age-specific analyses showed the same trends. Similar to a bimodal curve, the effect of education first increased then declined, and peaked at age 25–29 years. After peaking, fertility dropped to a minimum at age 35–39 years, and then increased, reaching another peak at age 40–44 years. The impact of education was not substantial in the groups aged 15–19 and 20–24 years. The age group most impacted by educational attainment in 1990 was women aged 35–39 years, and in 2000 and 2010 was those aged 40–44 years. The positive effect of education was increasingly obvious over time. Age 15–29 years is generally considered to be the most fecund period for childbearing among women, and a positive pregnancy outcome was more likely during these years regardless of level of education. After age 30 years, women’s fecund years have been occupied more and more, but before finishing fertility, the effect of education rebounded and formed a peak, indicating that women (especially educated women) still pursued an opportunity to give birth before substantial age-related fertility declined. The impact of women’s education on fertility is highly context-specific, and varies by time, region of the world, and level of development. Jejeebhoy (1995) suggested that education affects fertility in a non-linear fashion, with some education leading to somewhat higher fertility, but additional education lowering it.

Workforce experience showed a significant negative at age 25–34 years, but no effect in groups aged 15–24 and 35–49 years. There are a number of possible reasons for this finding. First, women’s workforce participation is affected by school attendance at age 15–19 years. Second, the opportunity cost of childbearing and childrearing may mean highly educated women delay family formation for several years after entry into the workforce, resulting in a postponement of childbearing. Third, some women work for a period of time before interrupting their career for motherhood. Considering the average length of women’s reproductive period, the impact of workforce experience on fertility was not significant for women over age 34 years. Women’s desire for motherhood typically presents a bell-shaped curve, gradually increasing at age 15–24 years, peaking at age 25–34 years, and then declining after age 34 years.

The variable *Pol* was significantly positive, ranging from 0.5 to 1 (standardized coefficients), meaning that the more loosely the One Child Policy was implemented, the more children were born. Under the One Child Policy, women who gave birth to their first child at age 15–24 years would be less affected at these later phases, with less conflict between childbearing and birth control policy. At age 25–44 years, women have experienced longer exposure to the birth control policy, while women aged 40–44 years would be considering their last opportunity for a child. The effect of the One Child Policy gradually declined as women’s age increased, especially among women at the end of their reproductive period. In places where the One Child Policy was implemented more loosely, more women of reproductive age may have wanted additional children. Under China’s current policy situation women’s desire for more children remains unfulfilled. At the same time, prolonged education may contribute to postponing childbearing until an age when biological factors may make it difficult to conceive (Kravdal 2001).

## Discussion

Our findings differ from those of previous studies. Billingsley (2011) found postponement of childbearing but no difference in birth rates across educational attainment groups in Russia. Huinink (2001) found a high percentage of childless women or women with two or more children among college graduates in West Germany, and Kravdal (2000) found no significant interaction effects between individual and aggregate education in Zimbabwe.

In summary, our results indicate that educational attainment, workforce experience, and the One Child Policy all impact on women’s fertility. The effect of education mainly occurred in women aged 25–29 and 40–44 years, whereas the main effect of workforce experience occurred in those aged 25–29 and 30–34 years. The effect of the One Child Policy covered various age groups. An important issue affecting the fertility decisions of women aged 35 years and older is their physiological status; whether or not they are able to give birth. Education and workforce participation put a time-squeeze on women’s fertility, causing many to postpone childbearing. Fertility decisions reflected both preference and timing factors, as women in Guangdong Province combined education, workforce experience, and childbirth. However, the overall impact of education appeared to be relatively low; as Edwards (2002) pointed out, only a fraction of the rise in age at first birth over the last 30 years can be explained by the increase in educational attainment. Moreover, the suppressed effect of women’s education through opportunity costs becomes smaller (Ermisch 1989).

## Conclusion and policy implications

Our analysis of census data from Guangdong Province brings a new perspective to the study of women’s fertility. We used cross-sectional age-specific comparisons to explore the effects of women’s education and workforce participation on fertility outcomes. Our main findings were: (1) under circumstances of low fertility, women tend to have more children with greater educational attainment; (2) the impact of women’s education and workforce experience on fertility varied across age groups, with the effect of education showing a bimodal curve peaking at 25–29 and 40–44 years, and the effect of workforce experience occurring at 25–34 years; and (3) the fertility time-squeeze effect by educational attainment was relatively small, the time-squeeze effect by workforce participation was larger, and the most important fertility time-squeeze effect was birth control policy and its implementation.

Age-specific estimates indicated an increase in women’s education reduced the length of their reproductive period, but increased their fertility desires. Workforce participation had a negative influence at age 20–49 years, and especially at ages 25–29 and 30–34 years. The effects of both education and workforce participation were not significant at age 19–24 years. Women’s fertility was greatest at age 25–34 years, as over age 34 years, childbirth decisions tend to be influenced by women’s physiological status. Health problems at later ages, often the result of infection, may also result in difficulty with ovulation and/or blockage of the fallopian tubes, making it more difficult for women to conceive.

The suppression of childbearing as the result of increasing education and workforce participation for women is unavoidable to some extent. However, together, the two factors reduce the length of women’s reproductive period, therefore reducing potential completed family size. Many women place high value on both family and career, and seek a balance between them (Kravdal 1994; Lappegård and Rønsen 2005). To increase the compatibility of education, work, and motherhood, the key issue is timing; first, when the woman’s education is completed and second, when the woman takes a break from her career to start a family. The solution to this timing problem depends on the net benefits for each woman, who must balance educational attainment, workforce participation, and family building. The present authors note that there is relatively little incompatibility when women in China have full-time work commitments as well as a child and family. The legal maternity leave in China is 98 days. Women also enjoy special protection during pregnancy, childbirth, and breastfeeding, as they are exempt from being fired. Therefore, most women do not quit their job at childbirth, and instead remain attached to the labor market.

With the aging of the Chinese population, the *demographic dividend* of economic growth has been reduced, and local governments now recognize the need for an ongoing supply of workers to enter the labor force. Among the policy alternatives suggested, relaxing the birth control policy is considered an effective way to meet workforce needs. The One Child Policy was adjusted in 2013, and a Two Children for Only-Child Couples Policy was trialed. This policy allowed couples (provided one was an only child) to apply for permission to have a second child. Further, on October 29, 2015, the Communist Party of China announced that China will abandon the One Child Policy and allow all couples to have two children. With the release of this constraint, it is likely that many women will consider having a second child. Keeping in mind the interactions of women’s education and workforce participation and their effects on fertility in China, new institutional arrangements such as paid maternity leave, paternity leave, and insurance should be designed, or former arrangements revised, to provide sufficient services for couples planning to increase the size of their family.

## Abbreviations

TFR: total fertility rate
ASFR: age-specific fertility rate
